# Some Nurses' Problems

**Published:** 1919-11-15

**Authors:** C. Seymour Yapp


### THE EDITOR'S LETTER-BOX.

[Correspondence on all subjects is invited, but we cannot in any way be responsible for the opinions expressed by our correspondents, who must give their name and address as a guarantee of good faith, but not necessarily for publication. Correspondents are reminded that brevity of style and conciseness of statement greatly facilitate early insertion.]

### Some Nurses' Problems.

*To the Editor of* The Hospital.

Sir,—Only too well do I realise that the sound ethical principle involved in the fact that any nurse taking an underpaid appointment perpetuates the evil, must inevitably be sacrificed to economic exigency under present conditions. But the principle is none the less true. In the instance referred to by "Dagmar," I rather hoped it would deter those already holding appointments from applying, and so reduce the number of applications. Unfortunately, many of these posts are being filled by women, not demobilised nurses, who cannot plead economic compulsion. It is nothing short of tragedy that fully-trained nurses who have seen service overseas should be forced to accept underpaid employment, or face the winter out of employment. To offer sympathy is futile; the thing to do is to organise and tackle our problems in the most effective way. With nearly 2,000 trained nurses out of employment, and probably quite as many semi-trained or untrained women, exploited by proprietors of nursing homes, is it to be wondered at that nurses in desperation argue bitterly, "If I do not take this underpaid post, another will." As a trained nurse of nineteen years' experience, may I say, with all the power at my command, in the College of Nursing I believe we have the finest nurse-organising machinery in the United Kingdom. And this is no light claim when it is said that the nursing profession is the most unorganisable and unorganised body of workers to-day. The College has now more than 16,000 members, all fully-trained nurses, who have paid their guinea membership fee because they believe it is the body best qualified to deal with their special problems. Apathy, and to a certain extent a certain fine sentiment, are responsible for our present economic position. We cannot afford to lose one iota of this fine feeling, but the time has come when the economic position of nurses must be seriously dealt with. The great problem for reformers is how best to adjust the economic position to modern standards without sacrificing the ideals which inspired our pioneers. Many of us honestly believe that the College can do this in the *best* way. May I earnestly plead with every trained nurse to think over the following?—

(1) If every one of us were united in the College of Nursing (as we all shall be one day!), what would be the position of employing bodies who offered salaries below the minimum laid down by our College? (It offers room for castle-building, doesn't it? Are *you* going to carry bricks to help it materialise?)

(2) If we wish for a Professional Union of Nurses, affiliated to the College, what is there to prevent it, so long as it is our common desire? Think it over, and to help you to understand what the College is doing, call for or send for the "Report of the Salaries Committee on Salaries and Conditions of Employment of Nurses" (6d.). This has been sent to all hospitals and has caused a flutter in the board-room of more than one, we hear.—Yours faithfully,

C. Seymour Yapp.

